# The prevalence of vitamin D deficiency among patients with type 2 diabetes seen at a referral hospital in Kenya

**DOI:** 10.11604/pamj.2019.34.38.18936

**Published:** 2019-09-17

**Authors:** Paul Bundi Karau, Bhatt Kirna, Erastus Amayo, Mark Joshi, Stanley Ngare, Geoffrey Muriira

**Affiliations:** 1 Kenya Methodist University, School of Medicine, Department of Internal Medicine, Kenya; 2 The University of Nairobi, Department of Clinical Medicine and Therapeutics, Nairobi, Kenya; 3 Kenyatta National Hospital, Department of Medicine, Nairobi, Kenya; 4 Kenya Bureau of Standards, Department of Testing, Kenya

**Keywords:** Vitamin D, diabetes, glycemic control

## Abstract

**Introduction:**

The prevalence of diabetes mellitus is rising at an alarming rate, calling for more insights into its pathogenetic mechanisms, and other factors involved in its progression. The prevalence of vitamin D deficiency is higher in diabetic compared to non-diabetic patients, and is associated with poor glycaemic control. This has not been documented among diabetic patients in Kenya. Aims: to determine the prevalence of hypovitaminosis D among type 2 diabetic patients at Kenyatta National Hospital in Nairobi, Kenya.

**Methods:**

We recruited type 2 diabetic patients on follow-up at Kenyatta National Hospital. Measurements of height, weight and waist/hip ratios were taken. We drew 6mls of peripheral blood to determine vitamin D, zinc and HbA1c levels.

**Results:**

A total of 151 participants were recruited, with 69.5% females and mean age of 58.2 years. Hypertension was found in 72.8% of the participants, and obesity in 37.7%. The mean HbA1c levels were 8.46%, and 62.9% had poor glycaemic control. The mean vitamin D level was 31.40ng/ml. Vitamin D deficiency and insufficiency was found in 38.4% and 21.9% of the participants respectively. We found a significant inverse correlation between vitamin D and glycaemic control (r = -0.09, p = 0.044) and vitamin D and BMI (r = - 0.145, p = 0.045).

**Conclusion:**

In this study population on long-term follow-up for diabetes, there was high prevalence of vitamin D deficiency. This forms a basis for further management of patients with poor glycaemic control. Further studies are needed to document the causal association between poor glycaemic control and vitamin D deficiency.

## Introduction

Type 2 diabetes mellitus, characterized by pancreatic β-cell dysfunction and peripheral insulin resistance is now highly prevalent in developing countries. It is associated with significant morbidity and mortality due to microvascular and macrovascular complications. Diabetes affects over 300 million people worldwide, and its prevalence is expected to double by 2030, with low and middle-income countries carrying up to 80% of the burden [1]. Therapies for type 2 diabetes have dramatically improved over the last two decades, but the rising burden calls for more insights into prevention and management of the disease. Lifestyle changes such as weight loss, dietary modifications, exercise and cessation of smoking have been shown to improve glycaemic control and reduce complications of type 2 diabetes [1]. Vitamin D receptors are present in the β cells of the pancreas and vitamin D has been linked to insulin secretion regulation [2]. Vitamin D levels may have an inverse relationship with HbA1C and low levels of vitamin D show a correlation to increased incidence of type 2 diabetes [3]. Vitamin D deficiency is as highly prevalent as diabetes in the general population. Emerging evidence suggests that vitamin D is involved in the aetiology and pathogenesis of diabetes mellitus. Several studies have implicated vitamin D deficiency in the development and progression of diabetes, while high plasma vitamin D is related to lower risk of developing diabetes in high risk patients. Vitamin D deficiency is involved in central pathogenetic mechanisms of diabetes mellitus; it affects insulin sensitivity and β-cell function [4]. In this study, we aimed to assess vitamin D levels among type 2 DM patients on follow-up in an outpatient clinic in a large referral hospital, and document the association between hypovitaminosis D and glycaemic control.

## Methods

This was a descriptive cross-sectional study, conducted among type 2 diabetic patients attending the diabetes outpatient clinic at Kenyatta National Hospital. The diabetes outpatient clinic runs once a week, and caters for 150 to 200 patients per week. It is manned by nurses, podiatrists, medical residents and consultants. Kenyatta National Hospital is a level 6 (National Referral Hospital) in Nairobi, Kenya, that handles referrals and specialized cases from all over the country. It has an inpatient bed capacity of 1500, and serves as a university teaching hospital for the University of Nairobi. We included all type 2 diabetic patients above 18 years of age who consented to the study. Patients were selected consecutively to join the study, until the sample size was attained. We excluded patients with renal disease, as evidenced by estimated glomerular filtration rate of < 30ml/min/1.73m^2^ calculated from serum creatinine, patients with liver disease (if Alanine Aminotransferase was > 5 times the upper reference limit) and patients taking multivitamin supplements, steroids, anticonvulsants (especially barbiturates and phenytoin). We also excluded patients who had taken multivitamins or vitamin D supplements within 6 months of the study. Kenya experiences two seasons (rainy and dry season), with the sun present in both seasons. This has little effect on Vitamin D in the bodies, hence no significant seasonal variations.

A pre-designed questionnaire was used to collect data on patient's age, gender, duration since diagnosis of diabetes, other co-morbidities, drug history, any diabetic complications and current treatment for diabetes mellitus. We performed anthropometric measurements of all eligible study participants in light clothing and without shoes. These included height, weight, hip and waist circumferences. Height and weight were measured to the nearest 10^th^cm and kg respectively. Body Mass Index (BMI) was calculated from the two measurements, in kg/m^2^. Hip circumference was taken as the greatest circumference at the level of greater trochanters (widest portion of hips) on both sides. The waist circumference was measured as the smallest horizontal girth between costal margins and iliac crests at minimal inspiration. The waist/hip ratio was calculated. Vitamin D was analyzed using High-Performance Liquid Chromatography (HPLC) method. We used the agilent 1100 HPLC system with a quartenary pump. Four milli-liters of blood were centrifuged and serum separated for use in the analysis. The concentration of vitamin D was measured in ng/dl, with ≥ 30ng/dl as sufficiency, 20.1-29.9ng/dl as insufficiency and < 20ng/dl as deficiency. It was extracted from serum using acetronitryl with 0.4% acetic acid. This was injected into a chromatographic column, Polaris C18-A 3μ 150 x 2.0mm. HPLC uses isocratic mode with eluent (MeCN: 0.4% Acetic acid) as the mobile phase. The flow rate was 0.3mls/min and oven temperature 30 degrees Celsius, and detector was UV-Vis at wavelength of 280nm. The amount of vitamin D was determined by matching the retention of pure standard and a calibration code. Data was coded, entered into SPSS version 23.0 (IBM), and cleaned. Continuous variables like age, BMI, Waist/hip ratio, vitamin D, were expressed in means ±SD, or median (Interquartile range). Categorical variables like vitamin D insufficiency and deficiency were analysed as proportions, n (%). Comparison and correlation of vitamin D levels and glycaemic control were done using paired students' t-test, with level of significance set at p < 0.05. Chi-squared tests were used to analyze relationships between categorical variables such as gender and vitamin D states (deficiency or insufficiency). We performed a bivariate analysis to see the relationship between vitamin D levels and glycaemic control. Ethical approval was sought from the University of Nairobi/Kenyatta National Hospital Ethics and Review Committee, and patients were consented accordingly (ethical approval reference number KNH-ERC/A/207).

## Results

**Sociodemographic characteristics of the study participants:** between July and December 2016, we recruited a total of 156 study participants. We excluded 5 because of incomplete records or inadequate blood samples. The mean age of the participants was 58.2 years (Interquartile range 50-67, median 58.0), with 105 (69.5%) females and 46 (30.5%) males. The sociodemographic features of the study population are further elucidated in Table 1.

**Table 1:** Socio-demographic characteristics of the study participants (n= 151)

Characteristic	n = 151
Age, (Mean in yrs, ±SD)	58.2 ± 12.2
**Gender (n, %)**	
Male	46 (30.5)
Female	105 (69.5)
**Marital Status (n, %)**	
Married	109 (72.2)
Single	11 (7.3)
Separated/divorced	6 (4.0)
Widowed	25 (16.6)
**Education level (n, %)**	
None	13 (8.6)
Primary	43 (28.5)
Secondary	64 (42.4)
Tertiary	31 (20.5)

**Clinical and anthropometric characteristics:** the mean duration since diagnosis of diabetes was 9.2 years. Majority had diabetes for more than 10 years (58, 38.4%). Most of the participants (72.8%, 110) were on follow-up for hypertension. The mean blood pressure was 143/82 mmHg. The commonest mode of diabetes treatment was a combination of insulin and oral hypoglycaemic agents, in 67 (44.4%) of the participants. The proportion of obese study participants was 78.8% (119) on Waist-Hip Ratio and 37.7% (57) on Body Mass Index. Table 2 illustrates the past medical, clinical and anthropometric characteristics of the study population.

**Table 2:** Clinical and anthropometric characteristics of the study population

Clinical characteristic	All study participants	
	N	%
**Smoking history**		
Never smoked	133	88.1
Stopped smoking	16	10.6
Currently smokes	2	1.3
Duration since diagnosis of DM (years)	Mean	Median
	9.2 ±6.9	8.0
**Duration since diagnosis of DM**		
Less than 1 year	12	7.9
1-5 years	45	29.8
5-10 years	36	23.8
More than 10 years	58	38.4
History of hypertension		
Yes	110	72.8
No	41	27.2
**Current diabetic medications**		
Insulin injections	29	19.2
Insulin and OHAs	67	44.4
OHAs only	48	31.8
No medications	7	4.6
**Body mass index**	Mean- 28.53	IQR
	Median-28.13	25.7-31.36
**BMI Category**		
18.5-24.9 (Normal)	31	20.5
25.0-29.9 (0verweight)	62	41.1
30.0-39.9 (Obese)	57	37.7
> 40 (Morbid obesity)	1	0.7
**Waist-Hip Ratio**	Mean- 0.91	IQR
	Median-0.90	0.86-0.94
**Waist-Hip Ratio category**		
<0.80 (Normal)	12	7.9
0.80-0.84 (Overweight)	20	13.2
> 0.85 (Obese)	119	78.8

OHA; Oral Hypoglycaemic Agents, IQR; Interquartile range

**Figure 1:**
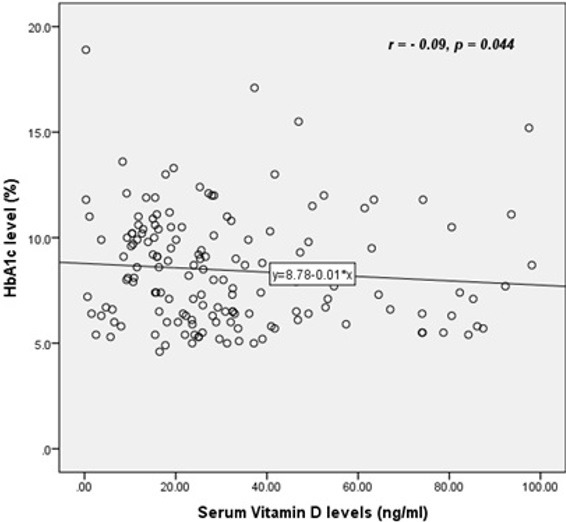
Scatter plot showing a negative linear correlation between HbA1c and vitamin D levels

**Figure 2:**
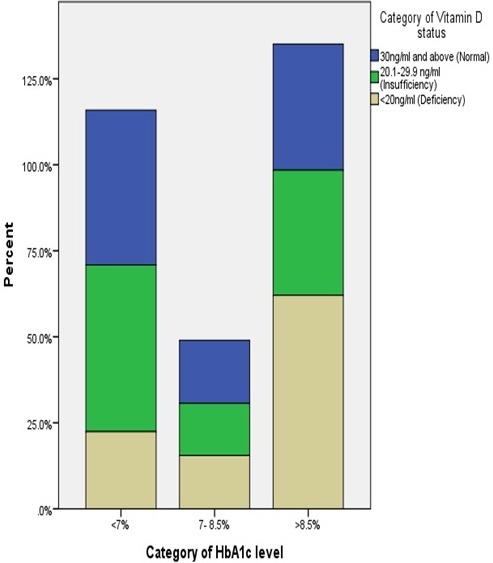
Stacked bar chart illustrating that patients with HbA1c > 8.5% were more likely to have vitamin D deficiency

**Biochemical characteristics:** the mean HbA1c level in the study population was 8.46 (±2.66). Majority of the participants (70, 46.4%) had HbA1c above 8.5%, indicating poor glycaemic control. The mean vitamin D level was 31.40 ng/ml (±23.22). We found Vitamin D deficiency in 58 study participants (38.4%). Table 3 illustrates the complete laboratory characteristics of the patients, while Table 4 shows the correlation between selected patient characteristics and vitamin D status. Bivariate analysis with Pearson correlation showed a significant inverse correlation between vitamin D levels and HbA1c (r = -0.09, p= 0.044), and between vitamin D levels and BMI (r = -0.145, p= 0.045). Figure 1 and Figure 2 illustrate the negative correlation between HbA1c and vitamin D levels.

**Table 3:** Laboratory characteristics of the study population

Laboratory Parameter	Mean ±SD (n = 151)	Median, IQR
Hba1c (%)	8.46 (±2.66)	8.00, 6.30-10.20
Vitamin D levels (ng/ml)	31.40 (±23.22)	25.36, 15.48-40.68
Categories of parameters	*N*	%
**HbA1c category**		
< 7.0% (Good glycaemic control)	56	37.1
7-8.5% (Moderate glycaemic control)	25	16.6
>8.5% (Poor glycaemic control)	70	46.4
**Serum Vitamin D**		
≥ 30ng/ml (Normal levels)	60	39.7
20.1-29.9 (Vitamin D insufficiency)	33	21.9
< 20ng/ml (Vitamin D deficiency)	58	38.4

**Table 4:** Correlation of selected characteristics with vitamin D status among the study participants

	Vit D deficiency (n, %)	Vit D insufficiency (n, %)	Normal Vit D (n, %)	Total (n, %)	P value
**Hypertension**					0.353
Yes	45 (40.9)	21 (19.1)	44 (40.0)	110(72.8)	
No	13 (31.7)	12 (29.3)	16 (39.0)	41 (27.2)	
**Duration of Diabetes**					0.337
≤ 5 years	21 (36.8)	16 (28.1)	20 (35.1)	57 (37.7)	
>5 years	37 (39.4)	17 (18.1)	40 (42.5)	94 (62.3)	
**HbA1c category**					0.012
≤7% (Optimal control)	13 (23.2)	16 (28.6)	27 (48.2)	56 (37.1)	
>7% (Poor control)	45 (47.4)	17 (17.9)	33 (34.7)	95 (62.9)	
**BMI of the patient**					0.660
Normal	12 (38.7)	4 (12.9)	15 (48.4)	31 (20.5)	
Overweight	22 (35.5)	16 (25.8)	24 (38.7)	62 (41.1)	
Obese	24 (41.4)	13 (22.8)	21 (36.8)	58 (38.4)	

## Discussion

**Prevalence of vitamin D deficiency:** the population in this study was on long-term follow-up for diabetes, with a mean duration since diagnosis of 9.2 years. The glycaemic control was largely suboptimal, with a mean HbA1c of 8.46, and only 37.1% with HbA1c of 7% and below. In this study, we found a prevalence of vitamin D deficiency of 38.4%, and insufficiency of 21.9%. This is lower that what is reported in type 2 diabetic patients in several studies conducted in Asia, Europe and North America. A study conducted in Saudi Arabia reported a prevalence of 59.8% of vitamin D deficiency and 38.6% insufficiency [5], meaning that over 98% of the study participants had suboptimal levels of vitamin D. In India, 32.1% and 34.6% of type 2 diabetics had vitamin D deficiency and insufficiency respectively [6]. Table 5 shows the prevalence of vitamin D deficiency among several study populations [5-11]. The lower prevalence of Vitamin D deficiency in our population could be partly attributable to climate; tropical countries experience greater and lengthier sun exposure compared to European, some Asian and American countries. Some of the differences observed in vitamin D levels across different populations are attributable to the cut-off levels adopted by the authors. There are no universally accepted cut-off levels for deficient, insufficient and sufficient vitamin D levels, although most studies have adopted < 20ng/ml, < 30ng/ml and > 30ng/ml for deficiency, insufficiency and sufficiency respectively. For instance, Kostoglou-Athanassiou *et al* [10] used a lower cut-off of 10ng/ml for deficiency, thereby possibly underestimating the number of subjects with deficiency.

**Table 5:** Prevalence of Vitamin D deficiency reported in previous studies among diabetic patients

Author	Population	% Vitamin D deficiency	% Insufficiency
Chiu *et al.* 2004	African Americans	54	Not done
Cigolini *et al.* 2006	Italians	60.8	Not done
Tahrani *et al.* 2010	Asians in UK	13	70
Zahrani *et al.* 2013	Saudi	59.8	38.6
Kostoglou-Athanassiou *et al.* 2013	Greeks	17.5	63.3
Palazhy *et al.* 2016	Indians	71.4	15
Akshay *et al.* 2017	Indians	32.1	34.6
Karau *et al.* 2017	Kenyans	38.4	21.9

The prevalence of vitamin D deficiency mirrors population vitamin D levels. In normal populations where the prevalence of vitamin D deficiency is high, the same is reflected, at a higher prevalence, among type 2 diabetic patients. For instance, in India, a study on vitamin D status in the normal population found a prevalence of 40-100% deficiency in the general population [12]. It is thought that the high prevalence of vitamin D deficiency in the general population in Middle East, India, Turkey and Pakistan is due to inadequate exposure to sunlight because adults, especially women, are covered in veils [12]. In Africa, low calcium in diet and frequent infections could account for the greater burden of Vitamin D deficiency. There are suggestions that skin colour may influence vitamin D levels. Chiu *et al* [7] reported racial differences in vitamin D deficiency, with 47%, 54%, 26% and 41% in Asian American, African American, White and Mexican Americans respectively. In Kenya, a normal population study found a mean vitamin D level of 65.5ng/ml (26.25-114.75) [13]. There is no study reporting the prevalence of vitamin D deficiency in the Kenyan population. Studies have however reported the prevalence of vitamin D deficiency in cancer and HIV patients. Among men with prostate cancer, vitamin D deficiency was found in 88.9% [14]. This high prevalence may be partly explained by the author's use of a higher cut-off of < 30ng/ml for deficiency, whereas we used a cut-off of < 20ng/ml. In a study among HIV-infected patients in Nairobi, 39% were vitamin D deficient, while 34% were insufficient [15]. These point to relatively high prevalence of vitamin D deficiency in the Kenyan population, although normal population studies are needed to elucidate the prevalence in the general population.

**Relationship between vitamin D and glycaemic control:** in the present study, a a significant negative correlation existed between vitamin D levels and glycated haemoglobin. A significant inverse correlation between glycaemic control and vitamin D levels has been reported by several authors [10, 16]. It appears that the inverse relationship is not limited to diabetes only, as Kostoglou-Athanassiou *et al* [10] reported a significant negative correlation between vitamin D and glycated haemoglobin in controls who were non-diabetic. On the other hand, some studies have found no significant correlation between vitamin D and glycaemic control [6]. A cross-sectional Finnish study found an inverse association between vitamin D levels and fasting insulin, fasting glucose and 2 hour glucose tolerance results [17]. This provides further evidence that low serum vitamin D may be associated with impaired glucose metabolism. An inverse association has been described between vitamin D levels and insulin resistance, especially at vitamin D levels between 16 and 36ng/ml [18]. Longitudinal studies have also reported a correlation between vitamin D levels and progression to overt diabetes among pre-diabetic and normal individuals [19]. A follow-up cohort of non-diabetic patients for 10 years revealed an inverse relationship between serum vitamin D levels and future glycaemic and insulin resistance [19].

There's no robust evidence on the role of supplementation of vitamin D on glycaemic control. One study conducted among African Americans showed that vitamin D supplementation significantly increased glycaemic control [20]. In one study, supplementation of vitamin D conferred a lower risk of diabetes to the participants when followed over time [3, 21]. The National Health and Nutrition Examination Survey (NHANES) III study (1988-1994) showed a strong inverse association between low levels of vitamin D and diabetes prevalence. The NHANES group study (2003-2006), which evaluated 9773 US adults above 18 years old showed a mechanistic link between serum vitamin D levels, glucose homeostasis and evolution of diabetes mellitus [22]. This study recommended that patients with elevated HbA1c levels should be evaluated for vitamin D insufficiency. The PRospective Metabolism and Islet cell Evaluation (PROMISE) cohort study examined the prospective association of baseline vitamin D with insulin resistance, beta cell function and glucose homeostasis in subjects at risk of type 2 diabetes. This study found that higher baseline vitamin D levels independently predicted beta cell function and lower AUCglucose at follow-up [23].

**Relationship between vitamin D and anthropometry:** the present study showed a high prevalence of obesity among the study subjects, with 37.7% in the obese category using BMI, and 78.8% using waist-hip ratio. This discordance is attributable to a very high prevalence of truncal obesity among the study population. Body mass index was inversely associated with vitamin D levels. Other studies have similarly reported an inverse association between vitamin D levels and body mass index, although the mechanism behind the relationship is not clear. In a study of 8421 participants from the National Health and Nutrition Examination Survey III (NHANES III), significantly lower levels of vitamin D were observed in the subjects with metabolic syndrome than in those without it [24]. Scragg *et al* [25] found that body mass index was significantly inversely correlated with serum vitamin D concentrations after adjusting for other covariates. A similar observation was reported by in a study conducted among Indian patients by Palazhy *et al* [11]. This inverse association between vitamin D and BMI is possibly connected to glycaemic control. There is robust evidence that poor glycaemic control is correlated with low vitamin D levels. Further, obesity is associated with poor glycaemic control, which may partly explain the low levels of vitamin D. Whether any mechanistic association exists between vitamin D and obesity remains to be elucidated.

**Study limitation:** our study cannot however show the causal association between glycaemic control and vitamin D levels. We therefore recommend further case-control and interventional studies.

## Conclusion

We found a high prevalence of vitamin D deficiency and insufficiency in our study population. The glycaemic control and body mass index correlated negatively with vitamin D levels. In long-term diabetics with poor glycaemic control, looking for other correlates of glycaemic control like vitamin D is prudent.

### What is known about this topic

- The relationship between vitamin D deficiency and poor glycemic control is well documented in previous studies.

### What this study adds

- This study has documented high prevalence of vitamin D deficiency in diabetic patients in Kenya;
- Further, this study shows a negative correlation between vitamin D and glycemic control, providing evidence for this mechanistic relationship in a Kenyan population.

## Competing interests

The authors declare no competing interests.
